# Late-stage rehabilitation effects do not differ between quadriceps and hamstring tendon autograft after anterior cruciate ligament reconstruction: a multicentre propensity score-matched case-control intervention trial

**DOI:** 10.5114/biolsport.2025.142647

**Published:** 2024-10-15

**Authors:** Daniel Niederer, Matthias Keller, Wolf Petersen, Natalie Mengis, Christian Eberle, Daniel Guenther, Georg Brandl, Björn H. Drews, Tobias Engeroff, Lutz Vogt, David A. Groneberg, Thomas Stein

**Affiliations:** 1 Institute of Occupational, Social and Environmental Medicine, Goethe University Frankfurt; 2 Department of Movement and Training Science, Faculty of Humanities and Social Sciences, Institute of Sport Science, University of Wuppertal, Wuppertal, Germany; 3 OSINSTITUT ortho & sport, Munich, Germany; 4 Klinik für Orthopädie und Unfallchirurgie, Berlin, Germany; 5 Arcus Sportklinik, Pforzheim, Germany; 6 KSA Aarau/Spital Zofingen, Switzerland; University Hospital, Basel, Switzerland; 7 Department of Orthopaedic Surgery, Trauma Surgery, and Sports Medicine, Cologne Merheim Medical Center, Witten/Herdecke University, Germany; 8 Department of Orthopaedic Surgery II, Herz-Jesu Krankenhaus, Vienna, Austria; 9 St. Vinzenz Clinic Allgäu, Pfronten, Germany; 10 Department of Sports Medicine and Exercise Physiology; Goethe University Frankfurt, Frankfurt am Main, Germany; 11 SPORTHOLOGICUM Frankfurt – Center for Sport and Joint injuries, Frankfurt am Main, Germany

**Keywords:** Propensity score, Autograft, Functional capacity, Return to sports, RTS, Intervention, Re-injury

## Abstract

Late-stage rehabilitation interventions after an anterior cruciate ligament (ACL) reconstruction are under-researched, inter alia regarding potential differences in rehabilitation effects between autograft types. This study determined the effectiveness of a specific, late-stage rehabilitation to usual care after ACL reconstructions in patients with a quadriceps versus such with a hamstring tendon autograft. In this multicentre case-control intervention study, participants aged 18–35 years were included at the end of their formal rehabilitation (mean 8.1 months) after ACL reconstruction. Twenty-four cases with an arthroscopically assisted, anatomic ipsilateral quadriceps femoris tendon autograft and two numerically equal hamstring tendon reconstructed propensity score-matched groups were compared. Matching variables were gender, age, Tegner activity scale, plus, once, the time since reconstruction and once the functional capacity at intervention onset. All participants performed a 5-month performance enhancing intervention. All outcomes were measured once a month. Self-reported outcomes such as knee function (The Knee injury and Osteoarthritis Outcome Score (KOOS) Sport as the main self-reported outcome) were followed by a series of hop and jump tests. The front hops for distance (outcome: hopping distance) was the primary outcomes of the study. Linear mixed models were calculated using change scores. All participants were analysed. No group*time interaction effect could be identified in the two main outcomes KOOS SPORT and front hop for distance. Furthermore, with the exception of the self-reported all-day function, no outcome displayed any between-group differences in the trainability, either. The return-to-sport success took a mean time of 3.8 months after study commencement; the success rates ranged between 80% and 83% and were not different between groups. Being reconstructed with a hamstrings or with a quadriceps tendon autograft had no impact on the late-stage rehabilitation effects after an ACL rupture. Both graft choices enable comparably favourable functional outcomes and return-to-sport success rates. Conversely, no recommendation can be derived with regard to the selection of either a hamstring or a quadriceps autograft type. The decision must be undertaken individually and based on other factors.

## INTRODUCTION

After an anterior cruciate ligament (ACL) rupture, numerous tendons such as patellar, hamstrings or quadriceps can be selected as autograft for a state-of-the art reconstruction [1]. Standard rehabilitation after ACL reconstructions leads to comparable graft survival rates and clinical stability results when semitendinosus (alone or in combination with gracilis) (= hamstring) and quadriceps femoris tendon autografts are compared to each other [2, 3]. Furthermore, clinical and even functional outcomes at and after standard care may not be different between these two graft types [2, 4]. On the other side, patients after ACL reconstruction using quadriceps tendon autograft migth experience less donor site morbidity compared to patients treated with hamstring tendon autograft [2].

Regaining functional performance capacities is a major goal after the ACL reconstruction [5–7]. These capacities can be modified by adequate exercises [8]. The outcomes of early and mid-stage formal rehabilitations are likely not to be different between graft types [9]. Following this initial and mid-phase, a continuation of the exercises may, however, be required for recovery completion. Up to date, the effects of these late-stage rehabilitation interventions after ACL reconstructions following the initial rehabilitation period are under-researched; this also applies for a between-graft comparison of the effects of late-stage rehabilitations [10].

General recommendations for late-stage rehabilitations consist of continuous explosive neuromuscular performance and movement quality deficits restoration [11]. Preliminary evidence on hamstrings tendon autograft reconstructed individuals indicates that such latestage interventions further improve neuromuscular performance capacities, such as the performance in the front hop for distance, to a slightly superior extent than usual care in the rehabilitation after an ACL-reconstruction. A comparison of late-stage rehabilitation effects between grafts is lacking so far.

As both the duration until formal rehabilitation completion and the functional abilities at the completion of the standard rehabilitation are highly variable between individuals [12], a between-graft comparison of cases to controls of, on the one hand, function, and, on the other hand, time since reconstruction seems adequate to compare the prospective trainability of persons with these two graft types.

The purpose of the present study was to derive evidence on between-graft comparative effectiveness of a focused late-stage rehabilitation after ACL reconstruction and formal rehabilitation completion. We hypothesise that the effectiveness of late-stage 5-month rehabilitation after ACL reconstruction and formal rehabilitation completion does not differ between persons reconstructed with an hamstrings tendon autograft and such with an quadriceps tendon autograft in terms of (1) functional capacities, (2) self-reported function, and (3) return to sport success rates.

## MATERIALS AND METHODS

### Design and ethical aspects

This propensity-score-matched case-control multicentre intervention study was conducted within the PReP project [13]. Approval was provided by the Ethics Committee of the Hessen Regional Medical Council (reference approval no. FF 104/2017). Date of the final approval of the study protocol was June 27, 2018.

Each participant provided informed oral and signed informed written consent for participating in the intervention study prior to enrolment. The participants were prospectively monitored and motivated to maintain the scheduled training frequency. We planned and performed the study in agreement with the Declaration of Helsinki (Version Fortaleza 2013). The project was pre-registered in the German Clinical Trials Register (DRKS, German Clinical Trials Register (DRKS): registration number DRKS00015313, drks.de; 1st. October 2018).

### Participants

Ten physicians from six medical centres screened all allocated patients diagnosed with an ACL rupture. Only individuals with an acute unilateral ACL rupture and having passed an arthroscopically assisted, anatomic ipsilateral quadriceps femoris or a hamstring tendon autograft (semitendinosus tendon or semitendinosus-gracilis tendons autograft for the comparator groups) ACL reconstruction were considered eligible. Secondary inclusion criteria were being between 18 and 35 years of age, having been engaged in any type of sport prior to the injury, and aiming to return to the previous sporting activity and level. Exclusion criteria consisted of a meniscus lesion with a diameter of > 2 cm, (2) a cartilage lesion > The International Cartilage Repair Society (ICRS) II°, previous musculoskeletal surgery of the uninvolved (contralateral) leg, leg malalignment > 5°, a multi-ligament injury pattern, severe post-operative complications such as graft failure or arthrofibrosis, re-injury, chronic inflammation of the musculoskeletal system, and pregnancy. Acute inflammation of the musculoskeletal system or muscle soreness were further exclusion criteria for the single measurement points, not for the whole study.

From the inclusion onwards, all participants were prospectively monitored during their individual early- and mid-stage rehabilitation until formal rehabilitation completion. At formal rehabilitation completion, the intervention started and the baseline assessment was conducted.

### Autograft selection

Prior to the study screening and inclusion, each individual’s autograft was selected based on current recommendations [1]. Increased rerupture risk (due to age or athletes in high-risk sports) for example, rather led to the selection of quadriceps tendon, whereas a medial instability was considered a relative contraindication for the use of hamstring tendons. Overall, biomechanical and individual criteria such as age, type of sport, and occupational activities were considered. When it seemed to be adequate to preserve the hamstring complex and, thus, to avoid damage to the knee flexor compartment [9], a quadriceps tendon autograft was usually selected. From a biomechanical point of view, small hamstring tendon sizes may not be suitable for a graft in certain patients [14].

### Sample size determination

The sample size estimation was based on the effects of the self-reported sport-associated function (The Knee injury and Osteoarthritis Outcome Score (KOOS)-SPORT) at a 1-year post-surgery followup [3]. The mean value of 70 (standard deviation 23) points in the quadriceps, and of 76 (standard deviation 16) points in the hamstring graft group leads, when adopted to a matched-pairs-design, to an effect size of d = 0.365. Thus, under a 5% alpha and a 20% beta error probability, at least 24 full datasets per group were needed to be included in the analyses if a significant result should be found.

### Matching

The quadriceps graft participants were recruited to only be included in the present study. To find matching partners for each quadriceps graft participant, we screened all participants who had a hamstring graft, who were randomised in the intervention group and who had completed the intervention in the project RCT [15]. For this purpose, a logistic regression propensity score, utilising a matching ratio of 1:1, was performed. The matching procedure was performed twice: first, with the time since reconstruction as one of the matching variables and then with the baseline functional values (hopping distance in the front hop for distance of the reconstructed side) as another matching variable. In both procedures, all other matching variables were held constant: gender, age and the Tegner activity scale pre-injury. As a result of this procedure, the two resulting comparator groups contained duplicate persons and the effects of the intervention should, therefore, not be compared to each other but only to the quadriceps group.

### Intervention

We followed the Consensus on Exercise Reporting Template (CERT) consensus for the reporting of the exercise interventions [16]. The onset of the intervention was individualised; all participants were prospectively monitored by (repetitive) phone calls until their formal rehabilitation was completed and the intervention started. A treating orthopaedic specialist, physiotherapist-, and a self-release for the training components by means of reporting physiological readiness were the primary criteria for the onset of measurements [17]. Participants were not involved in any other kind of the design, conduct, or reporting of the study.

All participants included in this study performed the intervention. The intervention consisted of a 5-month home-based exercise with a scheduled training frequency of three times per week and a duration of 30 minutes for each session. The programme included basic preventive strategies, dynamic balance, running exercises/agility exercises, self-perturbed postural control exercises, and strengthening (closed kinetic chain resistance and open kinetic chain resistance) exercises. In the functional enhancement and recurrence prevention continuum, plyometrics, hopping, and jumping exercises followed. The initial sagittal plane jumps were followed by other planes jumps, and completed with a change of direction and cutting manoeuvres including running and agility exercises. Progression was undertaken based on functional criteria, not time based. Details on the intervention components and progression can be found in the study protocol of the main trial (on hamstrings grafts only) [13].

Prior to the initiation of the home-based self-administered intervention, all participants received individual instructions for their individually – prescribed components of the intervention programme. All participants subsequently discussed and exercised their individual intervention with the instructors first and, subsequently, with their individual rehabilitation team (physiotherapists and/or athletic trainer). Afterwards, the home-based self-administered intervention started. All participants were progressively contacted by the instructors once a month for discussion of the components and their progression. The progression itself was monitored by the rehabilitation team and based on a stepwise progression model [8, 13].

In addition, all participants completed a detailed exercise log during their home-based training; the frequency, intensity, type and time of the exercises and of all further rehabilitations and (where applicable) sporting activities were prospectively reported following current recommendations [18]. More detailed, the type [rehabilitation, type of sports, exercise], frequency [times per week], and dose [minutes per week] during each exercise and sport session were reported.

Prior to the individual intervention start and until the individual intervention completion, all effect estimating outcomes were assessed once a month. The monthly measurements, thus, started at the individual intervention onset (baseline). Follow-up was at usually until 6 month post inclusion, but at least until 12 months post-reconstruction.

### Outcomes

All baseline, measurement time during or not during COVID-19-associated restrictions, sociodemographic, injury, reconstruction and pre-injury outcomes were first collected by a structured telephone interview at inclusion and from the surgery report.

All outcomes were assessed therapists- and assessor-blinded. First, a battery of questionnaires, followed by a series of hop and jump tests. All self-reported outcomes were collected in an online – survey on SoSciSurvey. Knee function and symptoms were reported using the Knee Injury and Osteoarthritis Outcome Score (KOOS) subscales. The subscale sport (SPORT) was the primary self-reported outcome to answer the hypothesis (2). Pain (PAIN), symptoms (SYMPTOMS) and activities of all daily living (ADL) were the other subscales included as outcomes. The participants then completed the return to sport after ACL injury questionnaire (ACL-RSI) to report the potential fear of loading their reconstructed knee during a sporting activity [19]. The Tegner activity scale, to measure the participant’s sporting activity level and return to sport (RTS) [20], and the Tampa scale of kinesiophobia (TSK) [19], to measure their fear of movement, followed. The volume, type, intensity, and duration of all rehabilitation/sport /exercise measures undertaken since reconstruction were further reported. When the same type and level of sport as that prior to the injury, irrespective of competition or training, was reached, the participant was treated as an “RTS-success” (yes/no at the time of the measurements). This return to sport success, simply dichotomised as yes versus no, was the outcome to answer the hypothesis (3).

After a specific warm-up (jumping jacks and steps), the Front hop for distance (i.e., single leg hop for distance) was performed [21]. The front hop for distance hopping distance was the primary outcome of the study. The front hop for distance (FHD) assesses (the quantity of) dynamic balance and single-leg-jumping performance ability. The participants hopped frontally as far as possible with the test leg and had to land in a controlled manner. The hopping distance was measuredtoe to toe (at take-off and landing). The hands did not have to be kept on the hips, but could be used for hop and landing control. The Drop jump screening test followed [22–24]. Participants take a bipedal hip-width stance on a box with a 32 cm target height. A bipedal drop jump follows: frontal step – drop – reactive jump with the shortest possible ground contact time. Here, the outcome was the normalised knee separation distance (percentage hip width in comparison to knee separation distance) at landing and at the jump’s reversal point [25]. For the sagittal plane landing quality rating, the Balance front hop test [26] was performed. The frontal plane landing quality rating was undertaken using the Balance side hop test [26]. For both Balance hops, the participants had to hop over a square on the floor with a 40x40 cm edge length with their hands on their hips. The end position after landing had to be kept for at least three continuous seconds. Two hops per trial and leg were performed: the better attempt was selected to be further analysed. Further details on the test conduction and measurement quality criteria can be found in the protocol [13]. The participants were educated on how to perform the jumps and hops. In cases of incorrect execution, the tests and, where needed, the instructions for the tests were repeated.

All hop and jump tests were performed by self-administration and filmed from a 3-metre distance frontal position. The videos were transferred using a safe form of big content transfer (PowerFolder Enterprise File Sync and Share; Germany) and were expert-rated using the investigator-blinded videos. [27].

### Statistical analysis

The alpha-error threshold was set at 5% for all inference statistical analyses. All analyses were performed using SPSS (Version 28, IBM SPSS, USA). We followed the CHAMP statement when designing and reporting the statistical analysis [28].

Following plausibility and outliers checking, all analyses were subsequently performed on an intention-to-treat basis. Imputation was employed assuming that missing data was missed completely at random. Multiple imputations used chained equations and a fully conditional specification with 40 iterations to produce asymptotically unbiased estimations of the data.

Baseline data were displayed as means and standard deviations, while the main outcome data were displayed as means and 95% confidence intervals of the change scores since baseline. Main inference statistical analyses were, again, performed using each outcome’s change scores since as the dependent variables. As inference statistical analyses, linear mixed models for repeated measures were calculated. The independent variable time effects were modelled as random effects, while the independent variable group and all covariates (potential confounders and effect modifiers) were modelled as fixed effects.

To determine potential between-group differences in the returnto-sport-success rates, Mantel-Cox Log Rank tests were performed based on the cumulative survival (RTS success at each time point, yes or no) curves.

## RESULTS

### Sample

**FIG. 1:**
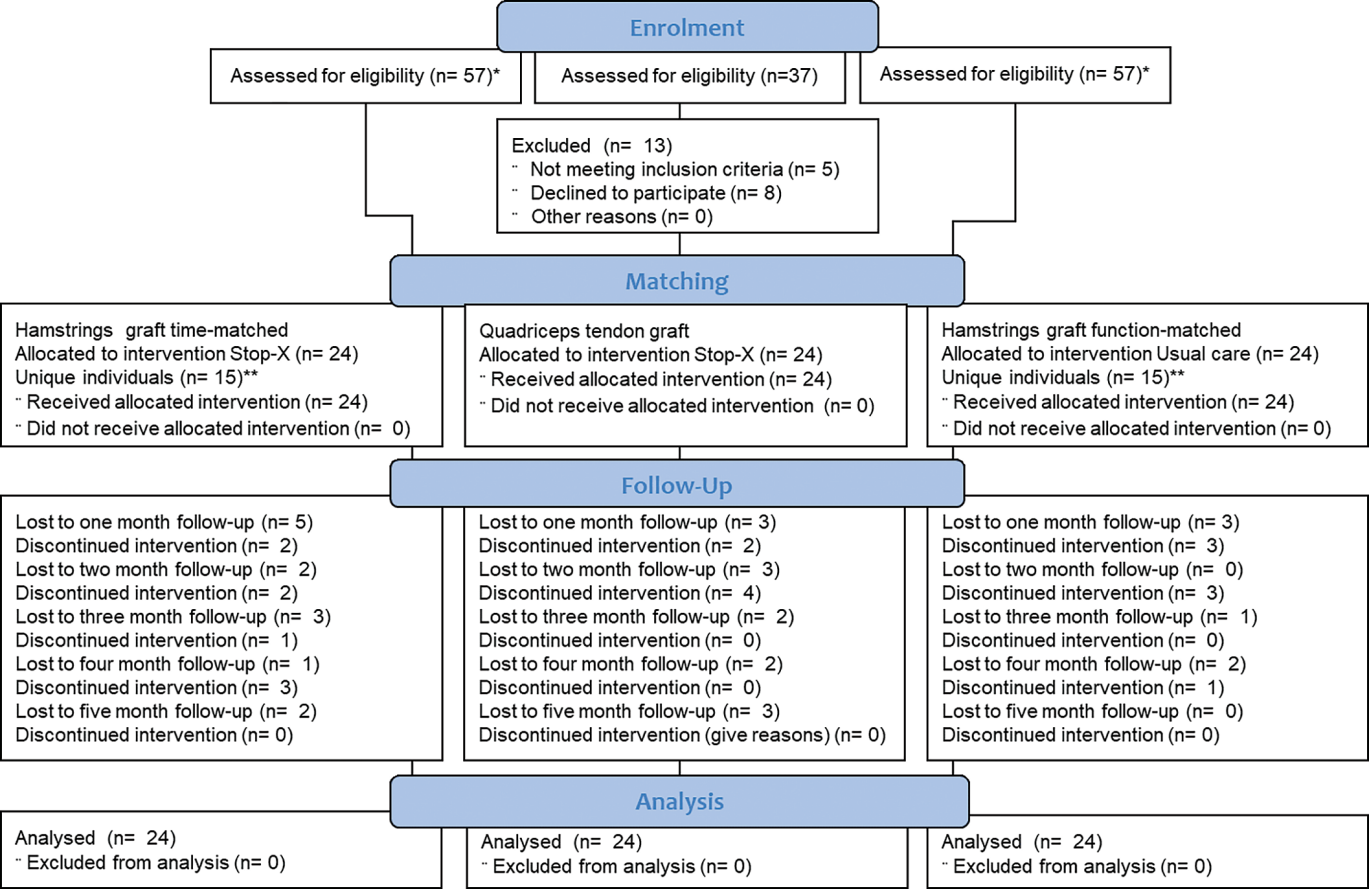
CONSORT participant flow. *the hamstrings graft participants sample from which the matching groups were selected, where the same for both matching groups. **thus, 9 participants were included in both comparison groups.

The quadriceps graft (case) sample consisted of 24 unique participants while the hamstring graft (control) sample comprised of 30 individual participants (Figure 1). Thus, there were 9 participants who were included in both hamstring graft control groups. Details on the Consolidated Standards of Reporting Trials (CONSORT) participant flow of the three groups are displayed in the flow chart in Figure 1.

Consent was withdrawn prior to the intervention due to a lack of time (from 13 participants), for reasons associated with the pandemic situation (6 participants), or no reason provided (from 7 participants). During the intervention, reasons given for discontinuation were a lack of time (4 participants), no reason provided or contact refused (8 participants), and the belief of being “healed” (2 participants). No serious adverse events occurred. Adverse events leading to exclusion included cyclops resorption (1 participant) and tendopathy (1 participant). In addition, adverse events leading to an interruption in the intervention or to a missed visit included job reasons (7 visits), secondary musculoskeletal pain (3 visits), acute infections (5 visits) and no reason provided (6 visits).

### Characteristics of study participants and baseline

Table 1 displays the baseline demographic and clinical characteristics for the total sample and for each group.

**TABLE 1:** Sample characteristics and baseline values. Numeric and percentage distributions of all baselines and traits: sociodemographic, sport-, injury- and surgery-specific characteristics of the study sample. The two control groups contained nine duplicate participants each. Categorical values are displayed as numbers (n) and percentage values (%), while the interval-scaled metric variables are displayed as means and standard deviations (SD).

A			Hamstrings time-matched		Quadriceps graft		Hamstrings function-matched	
Domain	Outcome	Value/unit	Numbers					
Socio-demographic	Gender ^$^	Female	12		12		12	
		Male	12		12		12	
		Other	0		0		0	

Covid-19: during restrictions?		No	4		15		2	
		Yes	20		9		22	

Domain	Outcome	Unit	Mean	SD	Mean	SD	Mean	SD

Socio-demographic	Body mass index	kg/m^2^	24.1	2.1	23.5	3.6	24.2	4.5
	Age ^$^	years	24.9	4.6	26.2	5.7	25.4	5.2

Time between…	injury and reconstruction	days	111.1	94.4	121.0	223.2	108.2	174.8
	reconstruction and study onset	days	243.6^$^	70.7^$^	266.2^$^	68.1^$^	219.4	57.0

**B**			**Hamstrings time-matched**		**Quadriceps graft**		**Hamstrings function-matched**	

**Outcome**	**Tool**	**Unit**	**Mean**	**SD**	**Mean**	**SD**	**Mean**	**SD**
Self-reported function	Tegner activity scale ^$^	points	5	2	5	2	5	2
	KOOS SPORT	%	76.8	10.7	65.3	24.9	73.6	14.0

Confidence	ACL-RSI	%	60.3	15.0	53.8	17.6	62.3	11.8

Kinesiophobia	Kinesiophobia (TSK)	points	23.0	3.3	21.4	4.7	23.2	4.9

Pain	KOOS PAIN	%	84.6	11.1	85.4	7.7	85.2	8.8

Knee symptoms	KOOS SYMPTOMS	%	74.0	13.8	76.4	14.3	73.7	11.3
Self-reported function	KOOS ADL	%	95.5	5.7	94.7	4.7	94.1	6.5

Front hop for distance	Reconstructed side	cm	96.3	21.3	86.9^$^	29.4^$^	95.9^$^	25.3^$^
	Contralateral side	cm	110.5	25.4	105.8	30.8	110.5	22.7
	Limb symmetry index	%	87.8	12.8	81.5	19.8	86.5	13.3

Drop jump	NKSD at landing	points	82.0	16.6	92.1	16.6	87.8	15.9
	NKSD lowest point	points	79.9	25.7	92.2	20.3	83.8	27.1
	NKSD transition	points	-2.11	14.1	0.12	13.2	-4.03	17.5

Balance side hop	Reconstructed side	points	3.8	0.94	3.72	1.46	3.81	1.1
	Contralateral side	points	3.64	1.2	3.65	1.38	3.73	1.11
	Side difference (absolute)	points	0.15	1.33	0.18	1.46	0.08	1.21

Balance front hop	Reconstructed side	points	3.89	0.97	3.94	0.85	4.21	0.94
	Contralateral side	points	4.31	0.83	4.06	1.18	4.04	1.04
	Side difference (absolute)	%	-0.42	1.13	-0.1	1.23	0.16	1.25

$ , matching variable; SD, standard deviation.

### Intervention dose and effects

**FIG. 2:**
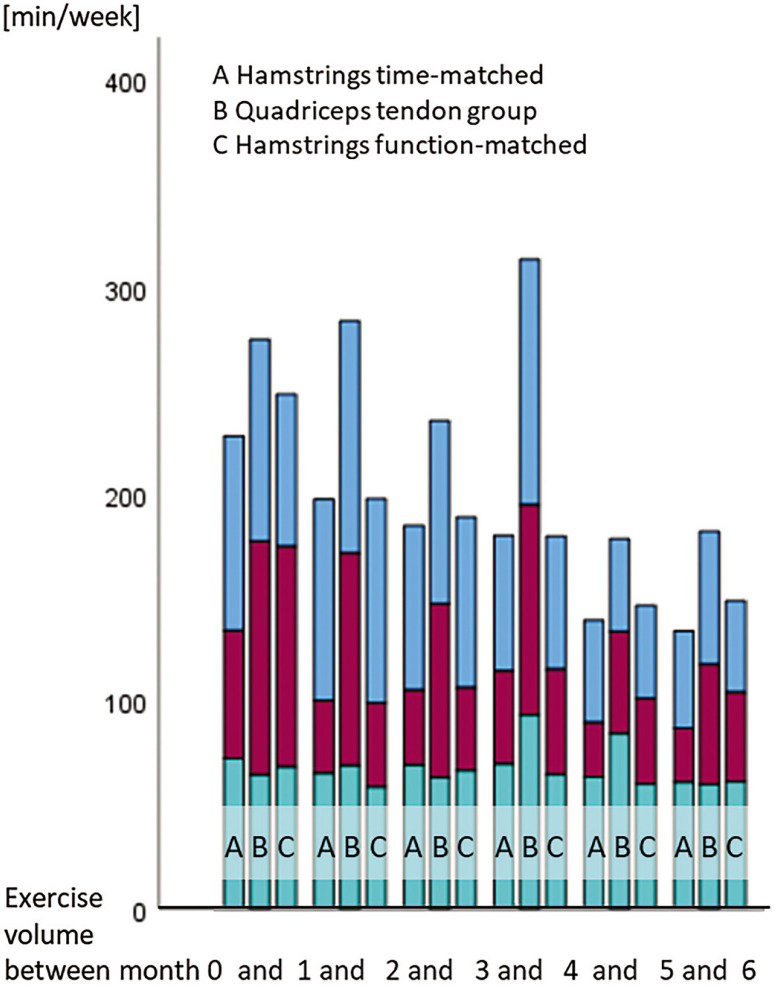
Training amounts for the graft type groups. The training amounts (in minutes per week) are separated into interventional measures, in formal/medically prescribed (lower portions of the bars) and self-administered rehabilitation measures (red, middle portions) and sporting activity (sport-specific training or competition, blue upper portions)).

The total training volumes were higher in the quadriceps graft than in the hamstrings graft groups. (Figure 2).

**FIG. 3:**
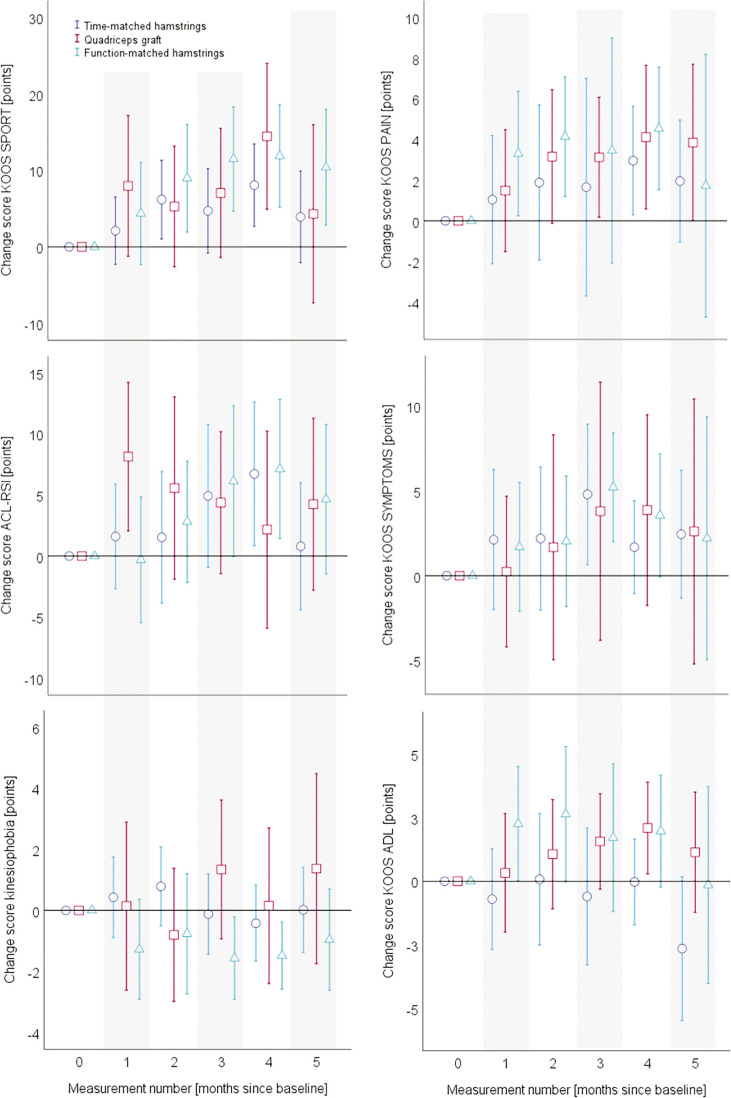
Mean values and 95% confidence intervals of the baseline-value-adjusted change scores for all patient-reported outcomes. Data are separated for the graft type groups. The x-axes always display the measurement number (month since baseline), while the y-axes show the values and confidence intervals for the respective outcome. KOOS, Knee Injury and Osteoarthritis Outcome Score; SPORT, sport sub-scale; PAIN, pain sub-scale; SYMPTOMS, symptom subscale; ADL, activities of daily living sub-scale; ACL-RSI, ACL-Return to Sport Injury Scale; TSK, Tampa scale of kinesiophobia.

**FIG. 4:**
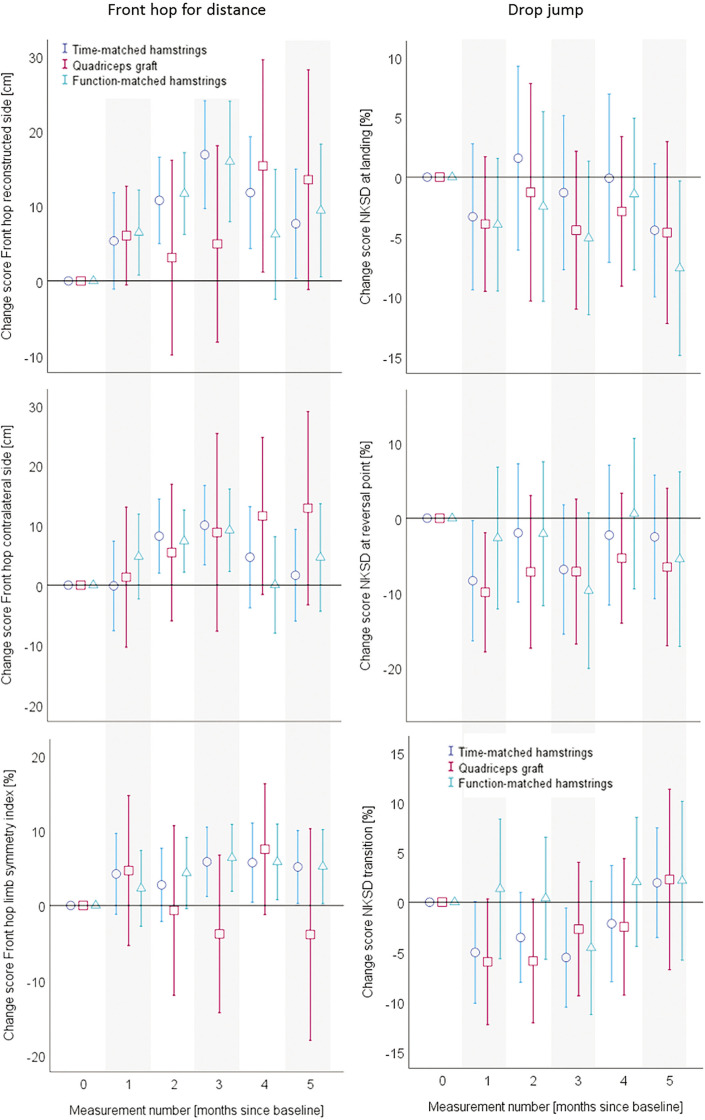
Mean values and 95% confidence intervals of the baseline-value-adjusted change scores for the front hop (left) and drop jump (right) outcomes. Data are separated for the graft type groups. The x-axes always display the measurement number (month since baseline), while the y-axes show the values and confidence intervals for the respective outcome. ACL, anterior cruciate ligament; NKSD, normalised knee separation distance.

**FIG. 5:**
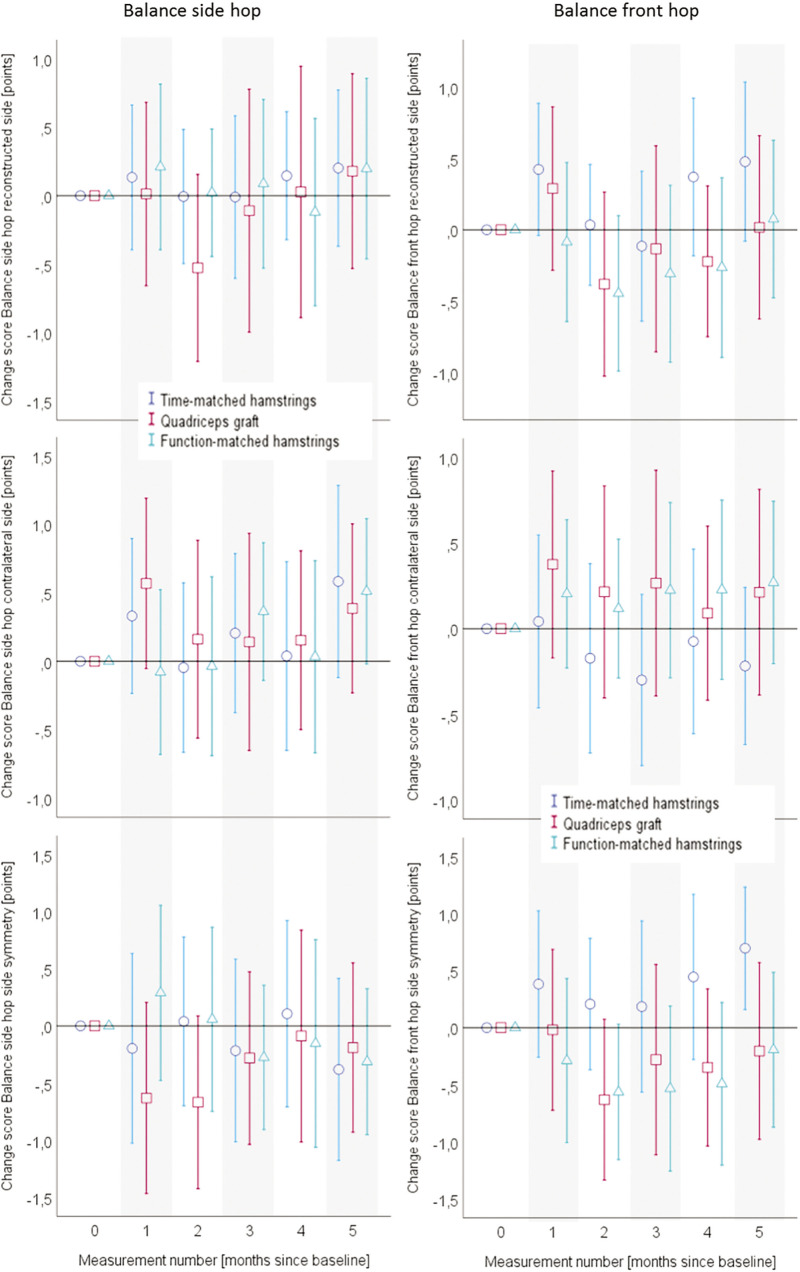
Mean values and 95% confidence intervals of the baseline-value-adjusted change scores for the Balance hops. Data are separated for the graft type groups. The x-axes always display the measurement number (month since baseline), while the y-axes show the values and confidence intervals for the respective outcome. ACL, anterior cruciate ligament.

Adjusted for baseline values, all patient-reported change-values are displayed in Figure 3. Figure 4 depicts the quantitative functional outcomes, whereas all the qualitative functional outcomes can be found in Figure 5.

The inference statistical analyses of the data displayed in the Figures 3 to 5 can be found in the Table 2. The main outcomes of the mixed model analyses are displayed as F-values, for all the self-reported and objective functional outcomes. All main effects (time, group), interaction (time*group) effects as well as the isolated and interactive contributions of the covariates are displayed. No group*time interaction effects could be identified in the two main outcomes of KOOS SPORT and front hop for distance occurred. However, a between-group*time effect occurred in the self-reported all-day function (KOOS ADL). Here, the participants with a quadriceps graft profited more from the intervention than those with a hamstrings graft. In all other outcomes, no betweengroup differences in the trainability occurred. In almost all outcomes, the baseline value had an impact on the group*time interaction.

**TABLE 2:** Main outcomes of the linear mixed models. F-values are shown; cases of significance are illustrated by means of an asterisk (*) to indicate significant contributors. For each outcome, the main effects (time and group) as well as the values for the interaction effects (time*group and time*group*covariate) are displayed. Part A displays the patient-reported outcomes, while part B shows the quantitative and part C the qualitative outcomes.

A	Self-reported function	Confidence	Kinesiophobia	Pain	Symptoms	Self-reported function

	KOOS-SPORT	ACL-RSI	TSK	KOOS Pain	KOOS Symptoms	KOOS ADL
Intercept	32.24*	7.83*	3.08	43.07*	21.07*	98.9*

Group	7.87*	0.38	0.83	2.23	4.46*	7.88*

Time: month of measurement	0.51	0.32	0.07	0.93	1.17	1.92

Baseline value	126.69*	24.9*	52.27*	100.44*	129.21*	144.94*

Gender	1.55	0.61	1.62	2.43	0.19	0.35

Pre- or during Covid-restrictions	0.48	0.32	1.51	0.14	5.99*	0.26

Tegner activity scale	0.0	0.01	0.51	1.78	2.17	0.01

Time between injury and surgery [days]	0.22	0.0	0.01	0.43	4.42*	1.53

Time between surgery and intervention start [days]	0.39	1.71	0.48	0.04	1.13	0.09

Total exercise rehabilitation [minutes]	0.35	2.11	3.26	7.41*	0.99	11.83*

Group*measurement month	0.75	0.44	1.0	1.0	1.12	2.23*

Group*measurement month*baseline	1.91*	0.83	1.1	1.91*	3.26*	3.88*

Group*measurement month*gender	0.66	0.82	0.92	0.42	1.08	0.88

Group*measurement month*Covid	1.66	0.96	0.85	0.84	1.49	0.64

Group*measurementmonth*Tegner activity scale	1.26	0.73	1.53	0.7	0.62	0.58

Group*measurement month*time between injury and surgery	0.68	1.14	0.75	1.32	1.58	0.71

Group*measurement month*time between surgery and intervention	1.0	0.41	0.92	0.59	0.8	0.55

Group*measurement month*total exercise rehabilitation	2.67*	0.56	0.73	1.19	0.82	2.82*

**B**	**Hopping ability (Front hop for distance)**			**Drop jump knee separation distance at…**		

	**Reconstructed side**	**Contralateral side**	**Limb symmetry index**	**landing**	**reversal point**	**transition**

Intercept	1.27	2.89	26.47*	16.56*	4.26*	4.26*

Group	2.29	0.34	0.53	0.24	0.9	0.9

Time: month of measurement	0.36	0.26	0.26	0.72	1.2	1.2

Baseline value	22.39*	51.36*	98.33*	212.9*	190.37*	190.37*

Gender	0.06	0.12	0.14	6.76*	19.74*	19.74*

Pre- or during Covid-restrictions	2.47	2.35	3.85	0.0	0.02	0.02

Tegner activity scale	16.74*	30.39*	0.0	1.91	4.84*	4.84*

Time between injury and surgery [days]	3.21	1.21	0.09	1.63	0.82	0.82

Time between surgery and intervention start [days]	7.88*	5.9*	0.33	4.44*	0.0	0.0

Total exercise rehabilitation [minutes]	2.04	1.51	0.51	0.43	1.69	1.69

Group*measurement month	0.33	0.12	0.68	1.57	0.88	0.88

Group*measurement month*baseline	1.79*	1.3	2.75*	1.01	0.99	0.99

Group*measurement month*gender	0.76	0.42	1.05	0.79	1.16	1.16

Group*measurement month*Covid	0.64	0.49	2.22*	0.84	0.9	0.9

Group*measurement month*Tegner activity scale	1.84*	1.53	1.45	1.22	0.76	0.76

Group*measurement month*time between injury and surgery	0.86	0.5	0.61	0.85	0.92	0.92

Group*measurement month*time between surgery and intervention	0.32	0.42	0.54	1.03	0.71	0.71

Group*measurement month*total exercise rehabilitation	1.89*	1.14	1.59	0.93	1.24	1.24

**C**	**Balance side hop**			**Balance front hop**		

	**Reconstructed side**	**Contralateral side**	**Side asymmetry**	**Reconstructed side**	**Contralateral side**	**Side asymmetry**

Intercept	13.37*	14.49*	0.01	19.75*	32.95*	0.09

Group	0.23	0.5	0.3	2.41	3.1	0.21

Time: month of measurement	0.7	0.69	0.73	0.47	1.1	0.65

Baseline value	181.74*	314.06*	277.8*	200.14*	298.86*	419.17*

Gender	0.07	1.7	0.77	8.78*	1.39	0.48

Pre- or during Covid-restrictions	2.26	0.45	0.91	0.01	0.08	0.56

Tegner activity scale	3.67	0.37	2.14	6.15*	0.03	2.29

Time between injury and surgery [days]	0.35	0.17	0.01	4.94*	0.29	4.45*

Time between surgery and intervention start [days]	0.39	0.01	0.96	2.31	3.67	1.66

Total exercise rehabilitation [minutes]	0.49	8.71*	3.49	1.12	2.69	0.02

Group*measurement month	0.46	0.41	0.55	0.47	1.0	0.43

Group*measurement month*baseline	1.24	0.7	0.49	0.99	1.57	0.65

Group*measurement month*gender	1.38	1.17	1.28	0.32	0.74	0.33

Group*measurement month*Covid	1.1	0.5	0.9	0.54	1.36	0.9

Group*measurement month*Tegner activity scale	0.57	1.28	1.12	0.53	0.6	0.59

Group*measurement month*time between injury and surgery	0.88	0.66	0.83	0.51	0.77	1.17

Group*measurement month*time between surgery and intervention	0.51	1.02	0.86	2.53*	0.84	3.71*

Group*measurement month*total exercise rehabilitation	1.47	1.55	1.04	0.79	1.88*	1.01

KOOS, Knee Injury and Osteoarthritis Outcome Score; SPORT, sport sub-scale; PAIN, pain sub-scale; SYMPTOMS, symptom subscale; ADL, activities of daily living sub-scale; ACL-RSI, ACL-Return to Sport Injury Scale; TSK, Tampa scale of kinesiophobia; NKSD, normalised knee separation distance; BSH, Balance side hop; BFH, Balance front hop; Covid, ongoing restriction associated with the Covid-19 pandemic; TAS, Tegner activity scale.

### Return to sport rates

**FIG. 6:**
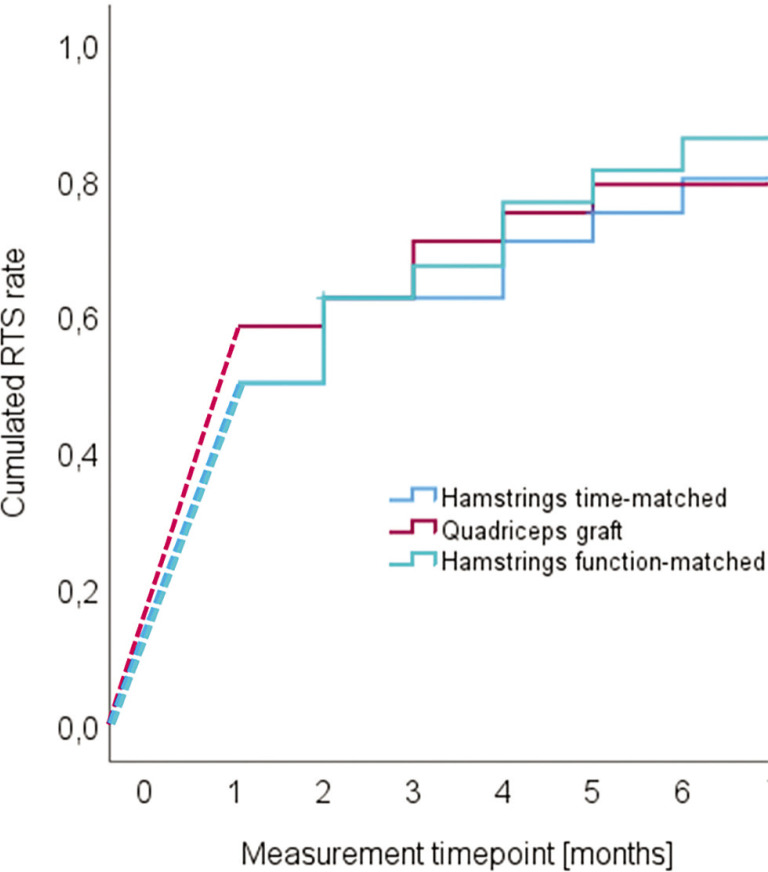
Cumulative return to sport- (RTS-)-success of the three matching groups.

The return-to-sport success rates per time were not different between the groups (Figure 6). The corresponding statistical criteria were Chi^2^ = 0.173 (p = 0.9). The overall mean RTS time was 3.8 months, always after the onset of the intervention [95% confidence intervals 3.3 to 4.4 months]. The total time until RTS thus lasted (mean) 11.8 month since reconstruction.

## DISCUSSION

### Statement of principal findings

In our case-control intervention study, we found that the late-stage functional trainability of hamstrings and quadriceps graft reconstructed individuals does not differ. Irrespective of the graft type, all groups showed improvements of self-reported and objective functional outcomes and return to sport rates over time in most of the assessed functional outcomes. No between-group differences, either, but also no improvements during the intervention conduction were found in the Balance hops and Drop jumps. All these results were independent of whether the hamstring control group was matched to the quadriceps graft case group in terms of time since reconstruction until or by function at baseline. Therefore, the hypotheses (1), (2), and (3) can be accepted.

### Comparison to the relevant literature

The between-graft type comparability after rehabilitation is, generally, in accordance with the current evidence [9, 29]. In the current literature no major between-graft differences [4] during and after formal rehabilitation are described for self-reported functional ratings, such as the IKDC [9, 29], Tegner-, or Lysholm-score [9], or in the objective functional outcomes, such as the Front hop for distance limb symmetry index [9] are described. This comparability between grafts could also be shown after a longer time period following the reconstruction; at a 2-year follow-up after ACL reconstruction, clinical and functional outcomes are likely not to be different between graft types [30]. The above sketched evidence was provided in standard and usual care settings. Thus, our results are expanding the current knowledge to late–stage rehabilitation. Likewise, in this late-stage setting, no between-graft differences in the trainability seems to be given. The small, but likely, additive benefit of the latestage rehabilitation, found in RCTs [31], is, thus, potentially valid irrespective of the graft type. In particular, with respect to a sufficient exercise volume prior to, and during, return to sport, in order to limit exposure to a too high acute to chronic workload ratio is of importance after formal rehabilitation completion. This importance provides a theoretical reasoning for the continuation of specific rehabilitation measures after early- and mid-rehabilitation completion and supports the effects found in the underlying randomised trial [15].

The differences in the interventional (time) effects on the functional outcomes may, inter alia, result from a ceiling effect in certain non-significant outcomes. For example, the baseline mean knee separation distance at the jump’s reversal point was 81.5%.; this value is far beyond the 60% cut-off value usually considered as the cutoff for a physiological drop jump [25]. The same may be true for the Balance hops: our participants’ values were, at baseline, already very close (mean = 4 points) to a “well/excellent” hop with a 5-pointrating [26]. Almost physiological movement patterns cannot improve much more.

Our return to sport-success rates generally fit with the relevant numbers in the literature. In the meta-analytic literature, mean success rates of 65% of non-elite athletes have been specified [32]. Our levels are somewhat higher and a better fit to the success rates of elite athletes. In elite athletes, a larger share of 83% to 100% return to their pre-injury type and level of sport [32–34].

### Contributors and effect modifier

The higher training amounts in the quadriceps than in the hamstrings groups may be a consequence of the COVID-associated restrictions. This might had further a potential impact on the duration until RTS. A larger share of the participants with a hamstrings graft than those with a quadriceps graft reconstructed ACL performed their rehabilitation measures during the pandemic situation. The restrictions were also associated with a reduction in the amounts of rehabilitation after ACL reconstructions [35].

In general, many further relevant factors beyond the graft type and the intervention type contribute to the functional outcome values after an ACL reconstruction. A dose-response relationships of the intervention, the time since reconstruction, time between injury and reconstruction, age, gender, pain, graft type, and concomitant injuries are not independent but are nested, interrelating predictors of functional outcomes after anterior cruciate ligament reconstruction [35]. The knowledge of their interactive contributions to motor function is helpful for the management of the reconstruction deficitoriented function-based rehabilitation and individualised return to sports strategies [36].

### Relevance for rehabilitation and graft selection

Important targets of the rehabilitation after ACL reconstructions are the restorations of the neuromuscular and motor knee-related functions [7]. To avoid a ceiling effect in late-stage rehabilitations, basic hop and jump tests may be continuously substituted by more sportspecific and problem-targeting measures, such as non-anticipatable jump-landings [37–39]. Performing long-term, late-stage-rehabilitation measures consisting of explosive neuromuscular performance and movement quality deficits restoration may be the most promising [11]. As found in our study, such an intervention may be feasible, and, although it is only slightly, effective. Given the comparable simplicity and low additional costs of such late-stage home-based self-conducted programmes, the small additional benefit over usual care [15] may already justify its usage in the late-stage rehabilitation after ACL reconstruction, may be also adoptable to quadriceps graft reconstructed knees.

Following the comparable results between grafts, the decision for selecting a hamstring or a quadriceps (or a bone-patellar-bone) tendon graft type cannot be undertaken based on our results solely. Considering the current literature, only the donor side morbidity is likely to be more severe in the hamstrings graft than in the quadriceps grafts [9, 29]. As reconstructions using quadriceps tendons are, for many surgeons and clinics, a rather new technique, a learning curve is, however, given: the re-rupture rate of quadriceps autografts appears to be strongly dependent on the surgeons’ experiences [40]. Nevertheless, in general, the graft selection decision must be undertaken individually.

### Methodological aspects and limitations

The late-stage rehabilitation measures adopted in our setting were all evidence-based and followed a stepwise function-based periodisation and progression [8, 12, 41]. Due to the multicentre approach and due to the local and health-assurance differences, minor differences regarding the design and structuring of the rehabilitation measures existed.

The reported training, rehabilitation and exercise amounts were considerably high. A certain risk for a reporting bias in the training protocol cannot be excluded. Furthermore, a common limitation in clinical exercise trials is the limited possibility of blinding the patients. This limitation was reduced by our case-control design in which both groups received the same trainings amounts. However, in a case-control approach, potential effects cannot, as it would be in a randomised approach, unambiguously be attributed to the intervention itself.

## CONCLUSIONS

A 5-month late-stage neuromuscular performance intervention programme after anterior cruciate ligament reconstruction may be feasible and effective in persons with a hamstrings and such with a quadriceps graft reconstructed ACL. Performing this late-stage rehabilitation may be promising to add a considerably smalsl, but additional, benefit to the usual care approaches in the late-stage rehabilitation after ACL reconstruction, irrespective of the graft type. This non-different trainability may also support the graft selection decision after ACL ruptures, based on the individual circumstances. Future studies are warranted to reproduce our findings in a randomised controlled context.

## Data Availability

The full dataset will be made available in a public repository when all project-wide studies and planned re-analyses are published. Until then, data are available upon request.

## ABBREVIATIONS

ACL, anterior cruciate ligament

CERT, Consensus on Exercise Reporting Template

DRKS, German Clinical Trials Register

ICRS, The International Cartilage Repair Society

KOOS, Knee Injury and Osteoarthritis Outcome Score

LSI, limb symmetry index

ACL-RSI, Return to Sport after Injury Scale

RTS, Return to Sport

TSK, Tampa scale of kinesiophobia
